# The Levels of Bone Alkaline Phosphatase (BALP) and Soluble Epidermal Growth Factor Receptor-2 (ECD/HER-2) in Pediatric Patients with Osteosarcoma During Clinical Treatment

**DOI:** 10.34763/devperiodmed.20182201.5864

**Published:** 2018-04-12

**Authors:** Magdalena Rychłowska-Pruszyńska, Joanna Gajewska, Jadwiga Ambroszkiewicz, Marek Karwacki, Katarzyna Szamotulska

**Affiliations:** 1 Department of Surgical Oncology for Children and Youth, Institute of Mother and Child, Warsaw, Poland; 2 Department of Screening and Metabolic Diagnostics, Institute of Mother and Child, Warsaw, Poland; 3 Department of Epidemiology and Biostatistics, Institute of Mother and Child, Warsaw, Poland

**Keywords:** bone tumor, prognostic marker, children, adolescence, guz kości, marker prognostyczny, dzieci, wiek młodzieńczy

## Abstract

**Aim:**

The aim of this study was to assess the usefulness of bone-specific alkaline phosphatase (BALP) and the extracelluar domain of human epidermal growth factor receptor 2 (ECD/HER-2) measurements in pediatric patients with osteosarcoma as prospective prognostic and predictive markers for monitoring the treatment and early detection of disease recurrence.

**Material and methods:**

We studied 22 patients (5 girls, 17 boys) aged 7-20 years with osteosarcoma (OS) treated at the Institute of Mother and Child in Warsaw. All the patients were evaluated for the serum levels of BALP and ECD/HER-2 before treatment, during pre- and postoperative chemotherapy and after the completion of treatment. Healthy children (n=22) were the reference group. The levels of BALP and ECD/HER-2 were measured using immunoenzymatic methods.

**Results:**

The values of BALP and ECD/HER-2 proteins were higher (p<0.01; p<0.05, respectively) in patients with osteosarcoma at the time of diagnosis compared with the control group. The values of both markers significantly decreased during chemotherapy in most patients with remission. In contrast to ECD/HER-2, the value of BALP after therapy was higher in patients with progression than with remission (p<0.001).

**Conclusions:**

Our results demonstrate the different pattern of BALP and ECD/HER-2 proteins during clinical treatment in patients with osteosarcoma. Higher values of BALP may characterize the progression of the disease and unfavourable prognosis. Further longitudinal studies are necessary to confirm the prognostic values of BALP and ECD/HER-2 proteins in this group of patients.

## Streszczenie

**Cel:**

Celem przedstawianego projektu była ocena przydatności oznaczania frakcji kostnej fosfatazy zasadowej (BALP) i zewnątrzkomórkowej domeny receptora ludzkiego epidermalnego czynnika wzrostu (ECD/HER-2) w surowicy krwi dzieci z kostniakomięsakiem (OS), jako potencjalnych markerów prognostycznych oraz predykcyjnych w monitorowaniu leczenia oraz jako markerów wczesnego wykrycia nawrotu choroby.

**Materiał i metody:**

Przebadano 22 pacjentów (5 dziewcząt, 17 chłopców) w wieku 7-20 lat z kostniakomięsakiem w Instytucie Matki i Dziecka w Warszawie. Wszyscy pacjenci byli oceniani pod względem stężenia BALP i ECD/HER-2 w surowicy przed rozpoczęciem leczenia, podczas chemioterapii przedoperacyjnej, pooperacyjnej oraz po zakończeniu leczenia. Zdrowe dzieci (n=22) stanowiły grupę kontrolną. Poziomy BALP i ECD/HER-2 mierzono metodami immunoenzymatycznymi.

**Wyniki:**

U pacjentów z kostniakomięsakiem w momencie rozpoznania wartości BALP i ECD/HER-2 w surowicy były wyższe w porównaniu z grupą kontrolną (p<0,01; p<0,05). Wartości obu markerów znacząco zmniejszyły się podczas chemioterapii u większości pacjentów z remisją. W przeciwieństwie do ECD/HER-2, wartości BALP po terapii były wyższe u pacjentów z progresją choroby niż z remisją (p <0,001).

**Wnioski:**

Nasze wyniki wskazują na różnicę w dynamice zmiany stężeń białek BALP i ECD/HER-2 podczas leczenia pacjentów z kostniakomięsakiem. Wyższe wartości BALP mogą charakteryzować progresję choroby i niekorzystne rokowanie u pacjentów. Dalsze badania są konieczne, aby potwierdzić wartość prognostyczną BALP i ECD/HER-2 w tej grupie chorych.

## Introduction

An osteosarcoma (OS) is the most common primary malignant bone tumor both in children and adolescents. In Poland 60-100 new patients with OS are diagnosed every year (the incidence rate is 2-3/1 000 000); 80% of those patients are in their 1^st^ or 2^nd^ decade of life. [1] Almost 20% of the patients present a disseminated disease at the time of diagnosis, and the most common site of metastasis are the lungs. In the past, treatment of OS consisted of surgery alone, and resulted in approximately 20% of event-free survival in patients with localized disease. Complex oncological therapy (COT) is still the treatment of choice in OS. The progress in COT, which currently resulted in the event-free survival of 60-70% of the children, relies mainly on chemotherapy, whereas radiotherapy is limited to palliative cases only. Till now no targeted therapy has been proven to be effective in OS. In more than 30 years no improvement in event-free survival rates has been observed.

The other problem of utmost clinical significance is to reveal the forthcoming progression or relapse of the malignancy in children with OS early enough to introduce appropriate treatment. The number of widely accepted biochemical prognostic markers in OS is limited to serum alkaline phosphatase (ALP) and lactate dehydrogenase (LDH) [2].

As a continuum of the previous works done in our Department [3, 4] and numerous publications worldwide [5, 6], we decided to find a biomarker of tumor progression in the form of a simple, reliable and easy biochemical test. We have measured the serum concentration of two markers: the bone-specific alkaline phosphatase (BALP) and the extracellular domain of human epidermal growth factor receptor 2 (ECD/HER-2).

The serum BALP level has been reported to play a role as a negative prognostic factor in OS, as well as a reliable and sensitive indicator for estimating tumor volume both in humans and dogs (absolute animal model of OS) [3, 5, 6]. The longitudinal observation of serum BALP concentration, especially when measured with other important biomarkers might – in our opinion – reveal the valuable marker for early diagnosis of tumor progression.

The expression of HER-2 in tissue and the level of its extracellular domain in serum have been found to be a helpful prognostic factor in many malignancies, especially in breast cancer [7, 8, 9]. Its role in OS is still controversial [10].

Based on the previous experience with both biomarkers in different malignancies, as well as our previous experiments, we decided to explore the clinical utility of the fluctuations of serum concentrations in children with OS.

## Aim of the study

The purpose of this project was to assess the usefulness of BALP and ECD/HER-2 values in blood serum of patients with OS in order to determine their usefulness as prospective prognostic and predictive markers for the monitoring of treatment and markers of early detection of disease recurrence.

## Material and methods

The study was carried out as part of the research project National Science Centre NCN N407 132939. We studied 22 patients (17 male and 5 female) aged 7-20 years (median age 15 years) with osteosarcoma, treated at the Institute of Mother and Child (IMiD) in Warsaw. The diagnosis of osteosarcoma, established by clinical and radiological findings, was confirmed histologically. The reference group consisted of 22 healthy children.

In 15 patients with OS, the primary lesion was located in the femur, in 4 in the tibia, and in 3 in the humerus. The size of the tumor in 14 patients was over 8 cm, and in 8 patients under 8 cm. In 9 patients lung metastases were observed at the time of diagnosis. Patients with osteosarcoma received preoperative chemotherapy (according to EURAMOS) composed of high-dose methotrexate, cisplatin, and doxorubicin. According to the protocol, after initial chemotherapy tumor resection was performed followed by histopathological examination. All the patients were operated on. Sparing surgery was performed in 19 patients and mutilating surgery in 3 patients. In the histopathological examination of the removed tumor, the dominant type was conventional (osteoblastic osteosarcoma in 12 patients, chondroblastic in 4, telangiectatic in 3 and other types in 3). 9 patients had a good response to initial chemotherapy (>90% necrosis), in 13 cases necrosis was <90%. Grade > GII was found in 18 patients, GI/GII in 4. Further chemotherapy was carried out in accordance with the EURAMOS protocol. Patients with poor response to treatment and with tumor necrosis <90% received intensified postoperative chemotherapy according to MAPI protocol. Patients with tumor necrosis >90% received chemotherapy according to MAP protocol. The metastasis resection was performed after regression or stabilization in the lung. All the patients were monitored at the Institute of Mother and Child after treatment ended.

All the patients were evaluated for the serum levels of BALP and ECD/HER-2 before treatment (biopsy), during pre- and postoperative chemotherapy and after termination of treatment. BALP activity was evaluated by a specific enzyme immunoassay utilizing a monoclonal anti-BALP antibody coated on the strip to capture BALP in the sample (BAP, Quidel, USA). The sensitivity of this assay is 0.7 U/L, intra-assay and inter-assay CVs are below 5.8% and 7.6%, respectively. Soluble ECD/ HER-2 protein concentrations were measured using the immunoenzymatic methods (Human sHER-2 ELISA, BioVendor, Czech Republic). The sensitivity of this assay is 0.06 ng/ml, intra-assay and inter-assay CVs are below 1.9% and 5.8%, respectively. Serum after centrifugation (1000xg at 4°C for 10 min) was stored at -20°C until biochemical analysis (no longer than 3 months). In order to exclude measurement errors, the assay was performed in duplicate and the mean of two consecutive measurements was used for statistical analysis.

Statistical analysis was performed using Statistica version 8.0. The Kolmogorov-Smirnov test and graphical inspections of data were used for evaluating distribution for normality. The results are presented as medians and interquartile range (25^th^-75^th^ percentiles) for non-normally distributed variables. Differences in biochemical parameters of patients were assessed using the non-parametric Mann-Whitney test for non-normally distributed variables. The p value of <0.05 was considered statistically significant.

## Results

In the entire group of patients with OS, overall survival was 77.2%, and progression-free survival was 63.73%. The follow-up period ranged from 12 to 47 months, median 37 months. Progression of the disease and recurrence occurred in 9 patients (40%). During treatment disease progression was observed in four patients, three of whom died. In 5 patients relapse of the disease was diagnosed over a period of 5 -24 months after the completion of treatment. In this group three patients were alive at the time of the submission of this article (out of whom one was in a state of remission), and 2 patients were dead.

BALP activity and serum ECD/HER-2 level in patients after biopsy following pre-operative chemotherapy, postoperative chemotherapy and values for the control group are presented in Table I. After tumor resection in patients with a good response, BALP activity was gradually increasing. Statistically significant differences were demonstrated in BALP and ECD/HER-2 values between OS groups (biopsy, pre- and postoperative) and the control group.

**Table I:** Serum levels of BALP and ECD/HER-2 in patients with osteosarcoma during treatment in comparison with healthy age matched counterparts. Tabela I. Poziomy BALP i ECD/HER-2 w surowicy krwi pacjentów z OS w trakcie leczenia w porównaniu z grupą kontrolną.

		Patients (n=22) *Pacjenci (n=22)*		Controls (n=22) *Grupa kontrolna (n=22)*
	Biopsy *Biopsja*	Pre-operative chemotherapy *Chemioterapia przedoperacyjna*	Post-operative chemotherapy *Chemioterapia pooperacyjna*	
BALP (U/L)	136.9 (85.7-240.6)	53.8 (40.4-84.8)^*^	63.8 (43.2-82.4)^**^	94.1 (59.4-120.4)^**^
ECD/HER-2 (ng/ml)	4.95 (3.53-12.3)	3.86 (2.79-10.08)^*^	3.84 (3.06-20.66)^*^	4.06 (3.29-5.20)^*^

Data are presented as median values and inter-quartile ranges (25^th^-75^th^ percentiles) *p<0.05 in comparison with patients with OS after biopsy **p<0.01 in comparison with patients with OS after biopsy *Dane zostały przedstwione w postaci median oraz przedziałów międzykwartylowych (25^th^-75^th^ procent)* *p<0.05 w porównaniu do pacjentów z OS po biopsji **p<0.01 w porównaniu do pacjentów z OS po biopsji

At the time of the diagnosis BALP and ECD/HER-2 values were compared in patients with metastatic and non-metastatic disease. There was an almost 3-fold higher serum BALP activity in patients after biopsy with metastases compared to patients without metastases (p<0.01). At each stage of the treatment, higher BALP activity was observed in patients with metastases than in patients with localized disease. No significant changes in ECD/HER-2 concentrations were observed, except for the increase of this marker level during postoperative chemotherapy in patients with localized disease (Table II).

**Table II:** Serum levels of BALP and ECD/HER-2 in osteosarcoma patients depending on the metastasis status at initial diagnosis. Tabela II. Stężenie BALP i ECD/HER-2 w surowicy krwi pacjentów z OS. Pacjenci zostali podzieleni na grupy w zależności od tego czy stwierdzono przerzuty na etapie dinagozy.

	Patients with localized disease (n=13) *Pacjenci bez przerzutów (n=13)*			Patients with metastatic disease (n=9) *Pacjenci z przerzutami (n=9)*		
	Biopsy *Biopsja*	Pre-operative chemotherapy *Chemioterapia przedoperacyjn a*	Post-operative chemotherapy *Chemioterapia pooperacyjna*	Biopsy *Biopsja*	Pre-operative chemotherapy *Chemioterapia przedoperacyjna*	Post-operative chemotherapy *Chemioterapia pooperacyjna*
BALP (U/L)	89.1(75.4-138.8)	43.0** (31.9-58.0)	50.5** (36.8-80.5)	257.0** (216.5-325.7)	101.6^*^ (49.7-142.6)	71.0^*^ (61.6-114.4)
ECD/HER-2 (ng/ml)	4.58 (3.40-14.20)	3.86 (2.86-12.9)	7.98^*^ (3.31-20.66)	5.11(3.68-6.90)	3.80 (2.44-5.76)	3.62 (2.59-6.88)

Data are presented as median values and inter-quartile ranges (25th-75th percentiles) ^*^p<0.05 in comparison with pre-operative chemotherapy in patients with localized disease ^*^p<0.05 in comparison with patients with metastatic disease after biopsy ^**^p<0.001 in comparison with patients with localized disease after biopsy ^**^p<0.01 in comparison with patients with localized disease after biopsy Dane zostały przedstwione w postaci median oraz przedziałów międzykwartylowych (25th-75th procent) ^*^p<0.05 w porównaniu do pacjentów z zlokalizowana chorob<a> podczas chemioterapii neoadiuwantowej ^*^p<0.05 w porówaniu do pacjentów z przerzutami po biopsji. ^**^p<0.001 w porówaniu do pacjentów z chorobg zlokalizawan<a> po biopsji. ^**^p<0.01 w porówaniu do pacjentów z chorob<a> zlokalizawan<a> po biopsji.

BALP activity was also analyzed in patients in remission, progression during treatment or recurrence of the disease. More than a 3-fold higher BALP activity was observed in patients with progression than in patients with localized disease (p<0.01). The difference in ECD/HER-2 concentration in patients with progression and without progression was not statistically significant (Table III).

**Table III:** Serum levels of BALP and ECD/HER-2 in osteosarcoma patients with remission or progression of disease after therapy. Tabela III. Poziomy BALP i ECD/HER-2 w surowicy krwi pacjentów z remisją lub progresją choroby po terapii.

	Patients with remission (n=16) *Pacjenci w remisji (n=16)*	Patients with progression (n=6) *Pacjenci z progresją (n=6)*
BALP (U/L)	43.6 (38.4-74.6)	147.0 (126.5-263.3)*
ECD/HER-2 (ng/ml)	3.56 (2.61-37.62)	6.41 (4.51-10.59)

Data are presented as median values and inter-quartile ranges (25^th^-75^th^ percentiles) *p<0.01 in comparison with patients with remission *Dane zostały przedstwione w postaci median oraz przedziałów międzykwartylowych (25^th^-75^th^ procent)* *p<0.01 w porówaniu do pacjentów z remisją

## Discussion

In the present study, changes in serum BALP levels in patients with OS during pre- and postoperative chemotherapy and after treatment were observed. The increase in BALP in patients with OS at the time of diagnosis appears to be associated with a poor prognosis, which has not been demonstrated for the second analyzed marker (ECD/HER-2). Previous studies conducted at our hospital indicate that BALP activity may be helpful in predicting prognosis and treatment monitoring [10].

In the work presented, the dynamics of changes of both parameters in patients with metastases and localized disease were analyzed at the time of diagnosis, during the subsequent stages of treatment and after the end of treatment. At the time of diagnosis, BALP was higher in patients with poor prognosis than in those with good prognosis. During chemotherapy, a decrease in the activity of this enzyme was observed, but in patients with poor prognosis the BALP serum level was higher than in patients with remission. In patients with progression of the disease the activity was 3 times higher than in patients with a good prognosis. These results are consistent with those of Liu et al. [12], who observed higher BALP activity in adult patients with osteosarcoma than in healthy subjects. Also, Zhiping et al. [13] showed significant differences in BALP activity in patients with malignant bone tumors in remission and progression. Levine et al. [14] found significant amounts of this de novo synthesized enzyme in human osteosarcoma cell cultures, especially in the osteoblastic sarcoma. It is known that BALP activity is associated not only with the osteogenic potential of the tumor, but also with tumor cell aggressiveness and metastasis. Numerous data indicate an increased BALP level in bone metastases [15].

The lower values of BALP seen in our study in patients during chemotherapy may be due to the effects of cytostatic drugs. Cytostatic agents (including methotrexate) used in the treatment of malignant bone tumors adversely affect intensive bone growth and mineralization during adolescence. In vitro studies have demonstrated inhibition of osteoblast proliferation under methotrexate metabolites [16]. This may result in a decrease in the value of bone turnover markers observed in this work and in earlier reports. [11] On the other hand, the higher values of BALP during chemotherapy in patients with poor prognosis may indicate an inadequate therapeutic effect of cytostatics. It could be that increased BALP activity in patients with bad prognosis may indicate the need to perform an additional diagnostic examination and possibly modify the therapy in these patients.

The half-life of the serum BALP marker is relatively long (1-2 days), so the variability of this parameter within a day is small (about 4%) [17]. However, it is important to remember that serum BALP activity is age-dependent and correlates with the growth rate, so the value of this parameter should be compared to the reference value obtained for the same age group. [18]

The HER-2 protein, encoded by the ERBB2 gene, is one of the four members of the epidermal growth factor receptor (EGFR) family. [16] Like its homologue, EGFR, HER-2 is a transmembrane tyrosine kinase receptor. [19] Investigations of HER-2 in osteosarcoma have led to the publication of numerous conflicting reports with regard to the level and prognostic value of HER-2 expression. [20] There are very few studies concentrated on researching the soluble part of HER-2 complex - transmembrane protein, also known as ECD- extracellular domain, in serum [4, 10].

Holzer et al. [10] found that serum levels of ECD/ /HER-2 in 27 patients aged 10-61 years with high-grade osteosarcoma, were no higher than in the control group (2.71 ng/ml in osteosarcoma vs 3.10 ng/ml in healthy individuals and 3.85 ng/ml in long-term survivors). This finding was irrespective of the histological subtype or grade, response to chemotherapy, metastasis or survival. Data was also statistically checked using Bonferroni’s principle. All simultaneous confidence intervals of soluble ECD/HER-2 overlapped, therefore no evidence of a difference in the means can be proven [10].

In contrast, Ługowska et al. [4] had shown a significant correlation between serum concentration of the ECD/ /HER-2 receptor and disease progression and overall survival. In this study conducted on 26 osteosarcoma patients the level of ECD/HER-2 in serum measured using the immunoenzymatic method before initial chemotherapy ranged between 3.8 and 14.4 ng/ml with a median of 5 ng/ml. Disease progression occurred statistically significantly more often in patients with ECD/HER-2 > 5 ng/ml (7/12 patients) than in patients with ECD/HER-2 < or = 5 ng/ml (1/14 patient). High concentration of ECD/HER-2 was also related to decreased overall survival: ECD/HER-2 >5 ng/ml vs ECD/HER-2 < or = 5 ng/ml: 50% vs 93%.

The current stage of knowledge cannot answer the question whether ECD/HER-2 measured in cancer mass or in serum can be used as a reliable marker for osteosarcoma. The differences in the results presented are caused by ununified methodology, different preparations of tissues used in research and different reactivity of mono- and polyclonal antibodies. Another issue is the lack of one set value which defines the expression of ECD/HER-2 as significant [4].

## Conclusions

Our results demonstrate different patterns of BALP and ECD/HER-2 proteins in patients with osteosarcoma during clinical treatment. We suggest that higher values of BALP may characterize the progression of the disease and unfavourable prognosis for patients with OS. Further longitudinal studies are necessary to confirm the prognostic values of BALP and ECD/HER-2 proteins in paediatric patients with OS.
